# New Remedy for Baldness

**Published:** 1881-07

**Authors:** 


### NEW REMEDY FOR BALDNESS.

In cases of confirmed baldness the new remedy proposed is to remove the scalp, bit by bit, and substitute, by skin grafting, pieces of healthy scalp, taken from the heads of young persons. The success which has heretofore attended operations of this nature in cases of scalp wounds gives a promising outlook for this new mode of curing baldness; and perhaps the day is not far distant when the shining pates of our venerable fathers will bloom with the flowing locks of youth.

